# A direct comparison of strategies for combinatorial RNA interference

**DOI:** 10.1186/1471-2199-11-77

**Published:** 2010-10-11

**Authors:** Luke S Lambeth, Nick J Van Hateren, Stuart A Wilson, Venugopal Nair

**Affiliations:** 1 Institute for Animal Health, Compton, Berkshire, UK; 2 Department of Molecular Biology & Biotechnology, University of Sheffield, Western Bank, Sheffield, UK; 3 Tumour Suppression Laboratory, Peter MacCallum Cancer Centre, St Andrews Place, East Melbourne, Australia

## Abstract

**Background:**

Combinatorial RNA interference (co-RNAi) is a valuable tool for highly effective gene suppression of single and multiple-genes targets, and can be used to prevent the escape of mutation-prone transcripts. There are currently three main approaches used to achieve co-RNAi in animal cells; multiple promoter/shRNA cassettes, long hairpin RNAs (lhRNA) and miRNA-embedded shRNAs, however, the relative effectiveness of each is not known. The current study directly compares the ability of each co-RNAi method to deliver pre-validated siRNA molecules to the same gene targets.

**Results:**

Double-shRNA expression vectors were generated for each co-RNAi platform and their ability to suppress both single and double-gene reporter targets were compared. The most reliable and effective gene silencing was achieved from the multiple promoter/shRNA approach, as this method induced additive suppression of single-gene targets and equally effective knockdown of double-gene targets. Although both lhRNA and microRNA-embedded strategies provided efficient gene knockdown, suppression levels were inconsistent and activity varied greatly for different siRNAs tested. Furthermore, it appeared that not only the position of siRNAs within these multi-shRNA constructs impacted upon silencing activity, but also local properties of each individual molecule. In addition, it was also found that the insertion of up to five promoter/shRNA cassettes into a single construct did not negatively affect the efficacy of each individual shRNA.

**Conclusions:**

By directly comparing the ability of shRNAs delivered from different co-RNA platforms to initiate knockdown of the same gene targets, we found that multiple U6/shRNA cassettes offered the most reliable and predictable suppression of both single and multiple-gene targets. These results highlight some important strengths and pitfalls of the currently used methods for multiple shRNA delivery, and provide valuable insights for the design and application of reliable co-RNAi.

## Background

Since the first application of DNA-delivered RNA interference (RNAi), the expression of short hairpin RNAs (shRNAs) for targeted gene silencing has become a benchmark technology. Using plasmid and viral vectoring systems, the transcription of double stranded RNA precursors that are processed by the RNAi pathway has lead to potent gene-specific knockdown. Importantly, such strategies can permit the long-term delivery of shRNAs to overcome the limitation of transient suppression by small interfering RNAs (siRNAs). Building upon the early experimental success of expressed shRNAs, the delivery of multiple RNAi effectors, known as combinatorial RNAi (co-RNAi), can offer considerable advantages over the use of single molecule knockdown strategies [reviewed in 1, 2]. Co-RNAi is particularly important for evolving targets that require long-term treatment such as highly mutable RNA viruses like human immunodeficiency virus (HIV) and hepatitis C virus (HCV). Recent studies have shown that the replication of these viruses can be suppressed for periods as long as 75 days by the expression of two of more shRNAs simultaneously [3-5]. In addition, the prospect of increased levels of gene silencing and for multiple-gene targeting is also extremely important for many other transcripts that are not particularly susceptible to spontaneous mutation such as host genes and DNA viruses.

There are currently three main methods to achieve co-RNAi in animal cells; multiple promoter/shRNA cassettes, long hairpin RNAs (lhRNA) and microRNA-embedded shRNAs. The expression of multiple shRNAs from a single construct encoding several separate promoter/shRNA cassettes offers the potential for relatively straightforward vector construction as previously validated RNAi cassettes can be simply assembled as tandem repeats. Recent studies have included the use of shRNA cassettes in combinations of two [6], three [3,7,8], four [4,9,10], six [11,12], and in one study a cloning strategy for the production of up to seven was described but not validated [13]. Results consistently show that such approaches provide an additive effect on single and multiple-gene knockdown on a variety of host and viral gene targets. Although in one study, individual shRNAs were transcribed at much lower levels when expressed from a 4 cassette construct compared to single copy vectors [4].

The use of lhRNA or extended shRNAs (e-shRNAs) represent a likely progression from single site targeting as long dsRNA are naturally processed as part of the RNAi pathway, and such molecules have been shown not to induce interferon mediated responses [14-16]. A number of recent studies have successfully utilised this approach to target and suppress HIV replication [5,15,17-21] and as a result, the parameters that determine efficient processing have been well defined. In particular, the effect of varying siRNA stem length and positioning, spacing between siRNAs stems, and the relative abundance of processed molecules have been tested [19,20]. Despite these advances, siRNAs have been produced from lhRNA precursors in a gradient with the most abundant and active being at the end distal from the loop, resulting in reduced silencing for the second, third and fourth siRNAs [5,19-21].

The insertion of shRNA sequences into naturally occurring microRNA (miRNA) precursor sequences represents a potentially favourable strategy as effector molecules should be processed and exported by the same cellular pathways as endogenous miRNAs. Moreover, it has been found that miRNA mimic shRNAs can abolish competition of siRNAs and shRNAs for transport and incorporation into RISC [22]. The insertion of an effective shRNA into the miR-30 pre-miRNA backbone sequence resulted in enhanced activity [23] and by embedding shRNAs in repeated miR-30 flanking sequences up to three shRNAs were transcribed from single constructs [24-27]. In addition, the BIC transcript which encodes miR-155, was modified to express multiple shRNAs [28] and a commercial vector featuring miR-155 flanking sequences and an shRNA cloning site has been widely used for shRNA delivery. The potential for multiple shRNA expression by modifying naturally occurring polycistronic miRNA clusters have also been shown using an endogenous human miRNA cluster [29] and chicken miRNA cluster [30].

Previous studies have clearly demonstrated that each co-RNAi strategy can be used to achieve highly effective gene suppression. However, these experiments generally focus on the use of one chosen co-RNAi approach, often involving substantial optimisation of that system. To provide an overall picture of the strengths and weaknesses of each method of co-RNAi, we took a set of active siRNAs and delivered them using validated and proven expression systems without extensive method-specific optimisation. By directly comparing the ability of these shRNAs to initiate knockdown of the same gene targets, this study provides practical information for researchers looking to express multiple validated siRNA sequences using an existing co-RNAi platform.

## Methods

### shRNA and lhRNA plasmids

All siRNA and shRNA sequences and their target reporter plasmids used in this study are listed in Table 1, and sequences of DNA oligonucleotides used to construct shRNA and lhRNA vectors are listed in Additional File 1. siRNAs were designed and synthesized by Eurogentec. Based on equivalent siRNA stem sequences, shRNAs and lhRNAs were constructed using annealed complementary oligonucleotides inserted into the BstBI and AscI sites of pchU6-3-ClaI vector as previously described [31]. This vector encodes the chicken U6-3 promoter inserted into the pGEM-T Easy cloning vector (Promega) and is a sequence that has been shown to transcribe shRNAs to similar levels as other chicken U6 promoters and the mouse U6 promoter (Wise et al., 2007; Kudo and Sutou, 2005). All 19-nt shRNAs used the miR-30 loop sequence (5'-CTGTGAAGCCACAGATGGG-3'), and lhRNAs were designed based on previously optimised lhRNAs [19] and used the loop sequence (5'-UUCAAGAGA-3') unless otherwise stated. All shRNAs and lhRNAs encoded a pol III termination sequence consisting of six thymidine residues. To construct the dual-U6/shRNA plasmids and the plasmids featuring up to five consecutive U6/shRNA cassettes, two backbone primer sequences were used: forward primer (chU6-F1: 5'-CCGCGGGAATTCGATTGACAACAC-3') and reverse primer (chU6-R5: 5'-CTTGAATTCATCGATGGGCGCG-3'), with various restriction sites added to each for different cloning steps. To construct the dual-U6/shRNA plasmids, shRNAs sh-1a, sh-1b and sh-2 were first cloned into pchU6-ClaI. Then, using chU6-F1 with an introduced SpeI site, and chU6-R5 with an introduced SpeI site, each cassette was amplified by PCR, digested with SpeI and inserted into the appropriate U6/shRNA plasmid also digested with SpeI. Plasmids featuring up to five consecutive U6/shRNA cassettes were cloned using a series of PCR and ligation steps (Additional File 2, Tables S1 and S2). Briefly, to construct shRNAx3, the U6/shRNA-3 cassette was amplified using chU6-F1 with introduced SbfI site and chU6-R5 with introduced SalI and NdeI sites, digested with SbfI and NdeI and ligated into dU6/F1a-F2 digested with the same enzymes. To construct shRNAx4, the U6/shRNA-4 cassette was amplified using chU6-F1 with introduced SalI site and chU6-R5 with an introduced MluI site, digested with SalI and MluI, and ligated into shRNAx3 digested with the same enzymes. To construct shRNAx5, the U6/shRNA-5 cassette was amplified using chU6-F1 with an introduced MluI site and chU6-R5 with an introduced NsiI site, digested with MluI and NsiI, and ligated into U6-shRNAx4 digested with the same enzymes. All plasmid and retroviral vectors used in this study were confirmed by DNA sequencing.

**Table 1:** siRNA and shRNA sequences and their reporter targets used in this study

siRNA	shRNA	Sequence (5'-3')	Reporter	Target gene (Ref)
si-1a	sh-1a	GCACAUUUGUCGAGCUUAA	psi-CHK-1	MDV gB [31]
si-1b	sh-1b	GGUUGGACAUGUACAAUAU	psi-CHK-1	MDV gB [31]
si-2	sh-2	GAGUUAUGCUGAUAUGAAU	psi-CHK-2	MDV UL29 [31]
si-3	sh-3	GGAGUUCACUGUAUCGUAC	psi-CHK-3	MDV ncRNA (unpublished data)
-	sh-4	GCUGGACUCCUUCAUCAAC	psi-CHK-4	*Renilla *luciferase (unpublished data)
-	sh-5	CAGCCAAUCACAUCCAUCAAA	psi-CHK-5	IBDV VP2 [47]
si-NS	sh-NS	UUCUCCGAACGUGUCACGU	-	-

### miRNA-embedded shRNA plasmids

**Figure 1:**
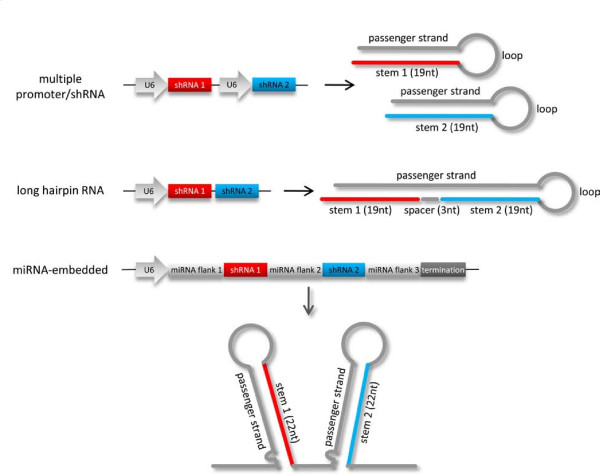
**Schematic representation of the strategies used for combinatorial RNAi**. Each method of co-RNAi results in the transcription of dsRNA precursor molecules that encode the siRNA stem (red or blue), a loop sequence and a passenger strand, that are processed into active siRNAs. The *top *panel shows a multiple promoter/shRNA construct that encodes dual U6 promoters to express two separate shRNAs simultaneously. The *middle *panel shows a long hairpin RNA (lhRNA) construct that uses a single U6 promoter to express an extended shRNA that encodes two separate siRNA stems. The *bottom *panel shows a miRNA-embedded construct that uses a single U6 promoter to express a miRNA cluster that features two introduced shRNAs that mimic naturally expressed miRNAs.

Sequences of DNA oligonucleotides used to construct the miRNA-embedded shRNA plasmids are listed in Additional File 1, Table S3. Vectors were constructed using the pRFPRNAi system as described previously [30]. The original 19-nt siRNA sequences were extended by 3-nt (22-nt stems) and featured an altered residue at the 5' base of each passenger strand to mimic the miR-30 structure (Figure 1). Briefly, hairpins for insertion into position 1 of pRFPRNAi were generated by PCR using gene-specific oligonucleotides along with the generic flanking oligonucleotides UHP1F+R. The resulting fragments were digested with NheI and MluI and ligated into pRFPRNAiC digested with the same enzymes. Hairpins for insertion into position 2 were then generated by PCR using gene-specific oligonucleotides and the generic flanking oligonucleotides UHP2F+R. These fragments were digested with MluI and SphI and ligated into pRFPRNAiC containing the relevant hairpin cloned in position 1.

### Retroviral vectors

shRNA, lhRNA and miRNA-embedded RNAi cassettes were cloned into the retrovirus RCASBP-(A)-CN-EGFP (with avian leukosis virus subgroup A envelope, a generous gift from Dr Jon Gilthorpe, Kings College London)[32] using the unique NotI site. Prior to the insertion of the dual-U6/shRNA cassettes into this vector, the chicken U6-4 promoter was amplified from chicken genomic DNA using U6-specific primers as previously described [33] with an introduced SpeI site in the forward primer (5'-GGACTAGTGAATTGTGGGACGGCGGAAG-3'), and reverse primer (5'-ATCGATGGGGCGCGCCGTTTAAACACTAGTTCGAACCCCAGTGTCTCTCGGACAGTA-3') with introduced BstBI, AscI and ClaI sites for the insertion of shRNA templates. Single U6/shRNA plasmids for the chicken U6-4 promoter were then generated for sh-1b and sh-2 using the same method as described previously [31]. These constructs were then digested with SpeI and ligated into U6-sh1a also digested with SpeI. The entire single and dual-U6/shRNA cassettes and U6/lhRNA cassettes were then excised by digestion with NotI and inserted into RCASBP-(A)-CN-EGFP also digested with NotI. To insert the miRNA-embedded shRNA cassettes, pRFPRNAi-specific primers were used to amplify the U6/miRNA inserts using forward primer (5'-TCGACCTGCAGCCCAAGCTT-3') and reverse primer (5'-ATAAGAATGCGGCCGCGCAGCGGATCCATCGATAAA-3') which contained an introduced NotI site. Amplified fragments were then digested with NotI and inserted into RCASBP-(A)-CN-EGFP that had also been digested with NotI. All U6/RNAi cassettes inserted into RCASBP-(A)-CNEGFP were in the reverse orientation.

### Reporter plasmids

**Figure 2:**
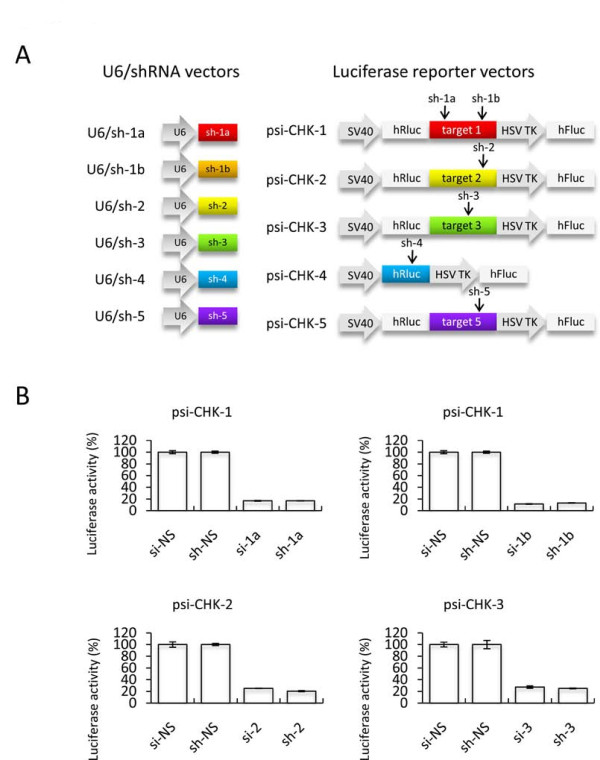
**shRNA vectors and their reporter targets, and the efficacy of siRNAs compared to equivalent molecules expressed as shRNAs**. (*A*) Schematic representation of the single U6/shRNA constructs and their target Luciferase reporter vectors used in this study. A total of 6 different shRNA sequences were cloned downstream of the U6 promoter. Using psi-CHECK-2, five different reporter vectors were constructed that encode Firefly luciferase and each of the shRNA target sequences fused to *Renilla *luciferase (except psi-CHK-4 as Renilla luciferase was the shRNA target sequence). For each reporter vector, the shRNA that targets each is indicated. (*B*) Normalised ratios of the Renilla:Firefly luciferase activity when DF-1 cells were co-transfected with the indicated reporter plasmids and either 50 nM of siRNAs or 125 ng of shRNA plasmid DNA. Values for siRNAs are shown as percentages of the negative control siRNA (si-NS), and values for shRNAs are shown as percentages of the negative control shRNA (sh-NS), as the mean of 4 replicates ± standard error.

All reporter plasmids were generated using psiCHECK™-2 (Promega) by PCR amplifying target sequences and inserting these into the NotI and XhoI site downstream of the *Renilla *luciferase gene (Figure 2A). The following primer pairs and template DNAs were used: psi-CHK-1; forward primer (5'-CCGCTCGAGTCCAAATCGCATCATATTAGGA-3') and reverse primer (5'-ATAGTTTAGCGGCCGCGCAAAATTTCCCGATCTTCTAG-3'), psi-CHK-2; forward primer (5'-CCGCTCGAGCGCTTTTACTCCTGCGGCAGAAACTA-3') and reverse primer (5'-ATAGTTTAGCGGCCGCGACAGCAACCAATGCCGAAATT-3'), psi-CHK-3; forward primer (5'-CCGCTCGAGGAGCGGTTTTTCTCCTTCC-3') and reverse primer (5'-ATAGTTTAGCGGCCGCGAACGAAGGGTTCCGATACA-3') were all amplified using MDV BAC pRB-1B5 DNA [34]. For psi-CHK-5, the infectious bursal disease virus (IBDV) VP2 gene cloned into pCI-Neo (Promega), named pCI-VP2, was a generous gift from Heba Mahgoub, Institute for Animal Health (Unpublished data), and was digested with NotI and XhoI and inserted into psiCHECK™-2 digested with the same enzymes. The psi-CHK-4 plasmid used was unmodified psiCHECK™-2, as *Renilla *luciferase was targeted by sh-4 (Table 1).

### Cells and transfections

The DF-1 cell line derived from line 0 chicken embryonic fibroblasts [35] were maintained in Dulbecco's modified Eagles medium (DMEM) containing 10% foetal calf serum with 10% CO_2 _at 39°C and used for reporter assays and for retrovirus growth. Plasmid DNA and siRNA transfections were carried out in 96-well plates using 125 ng of each plasmid DNA or 50 nM siRNAs using Lipofectamine 2000 (Invitrogen) following the manufacturer's instructions. For retrovirus growth, 2 μg of RCAS-U6/shRNA plasmid DNA was transfected into DF-1 in 6-well plates and passaged 6 days post-transfection into T75 flasks for RNA isolation or 96-well plates for reporter plasmid transfection.

### Reporter assays

The reporter vector psiCHECK™-2 carrying the sequences of the various shRNA target transcripts were assayed for luciferase expression using the Dual Glo Luciferase Assay System (Promega) following the manufacturer's instructions. The relative expression of target specific *Renilla *luciferase was determined by taking the normalised levels compared to background Firefly luciferase for each sample transfected in four replicates ± standard error and is representative of at least two independent experiments.

### Northern blotting

Total RNA was extracted from cultured DF-1 cells infected with retroviral vectors using TRIzol reagent (Invitrogen) according to standard methods described by the manufacturer. Samples of 30 μg total RNA were resolved using a 15% polyacrylamide-1 × Tris-borate-EDTA-8 M urea gel and blotted to a GeneScreen Plus membrane (Perkin-Elmer). DNA oligonucleotides with sequences complementary to the shRNAs were end labelled with [γ-^32^P] ATP (Amersham) and T4 polynucleotide kinase (New England Biolabs) to generate high-specific-activity probes. Hybridization, washing, and autoradiography were carried out as previously described [36].

### Statistical Analysis

Experimental data was analysed for statistical significance using two-tailed unpaired T-tests, where P values of less than 0.05 were considered significant. Statistical analysis was carried out using GraphPad Prism version 5.0b.

## Results

### Efficacy of single and double-gene targeting U6 expressed shRNAs

The simultaneous expression of several RNAi effectors can be achieved using various approaches, including multiple promoter/shRNA cassettes, long hairpin RNAs (lhRNA) and microRNA-embedded shRNAs (Figure 1). For the direct comparison of expressed shRNA from each co-RNAi platform, it was important to first test that the set of already validated siRNA sequences could be expressed as standard 19-nt shRNAs to achieve similar levels of gene knockdown. The sequences of all siRNAs and their target genes are detailed in Table 1, and all shRNAs and their target reporter plasmids are shown in Figure 2A. Co-transfection of reporter plasmids with either siRNAs or their equivalent shRNAs showed that for each of the four different molecules tested the efficacy of the expressed shRNA was very similar to its equivalent siRNA (Figure 2B), indicating that they were appropriate for use in the comparison of co-RNAi methods.

**Figure 3:**
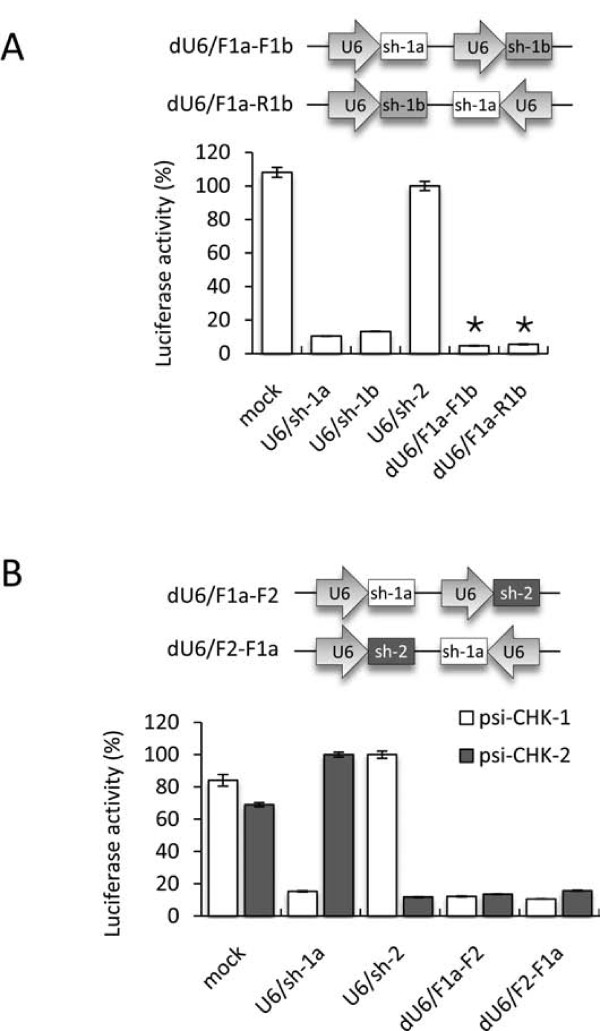
**Inhibition of Luciferase reporters by dual-U6 promoter delivered shRNAs**. (*A*) Schematic representation of the single-gene targeting dual-U6/shRNA plasmids, and normalised ratios of the Renilla:Firefly luciferase activity when DF-1 cells were co-transfected with psi-CHK-1 and the indicated RNAi plasmids (asterisks indicate P < 0.05 compared to equivalent single shRNAs, n = 4, two-tailed unpaired T-tests). (*B*) Schematic representation of the double-gene targeting dual-U6/shRNA plasmids, and normalised ratios of the Renilla:Firefly luciferase activity when DF-1 cells were co-transfected with either psi-CHK-1 or psi-CHK-2 and the indicated RNAi plasmids. The mock control refers to a reporter plasmid alone transfection. Values are shown as percentages of the non-targeting shRNA as the mean of 4 replicates ± standard error.

To test if single-gene targeting dual-U6 promoter constructs could provide additive gene suppression and to assess the impact of promoter orientation, the activity of plasmids with one U6/shRNA cassette in a forward orientation and a second cassette in either orientation were compared (Figure 3A). Co-transfection of these plasmids along with psi-CHK-1 showed that both dual-U6/shRNA vectors induced significantly greater reporter knockdown compared to the corresponding single shRNA vectors U6/sh-1a and U6/sh-1b (P < 0.05). In addition, since both dual-U6/shRNA were equally effective, these data suggest that the orientation of the second promoter does not considerably affect shRNA transcription. To assess if the location of each promoter sequence effected shRNA transcription, we then constructed double-gene targeting dual-U6/shRNA expression vectors. These contained two promoters in the forward orientation and encoded shRNAs targeting different genes in either the first or second positions (Figure 3B). Co-transfection of these plasmids with either psi-CHK-1 or psi-CHK-2 showed that both were able to suppress their corresponding targets similarly to equivalent single shRNA constructs and were equally effective in either of the two promoter positions. These data suggested that promoter location does not have an obvious impact on transcription efficiency and that each promoter can transcribe shRNA as effectively when present in plasmids with either one or two copies.

### Efficacy of single and double-gene targeting U6 expressed long hairpin RNAs

**Figure 4:**
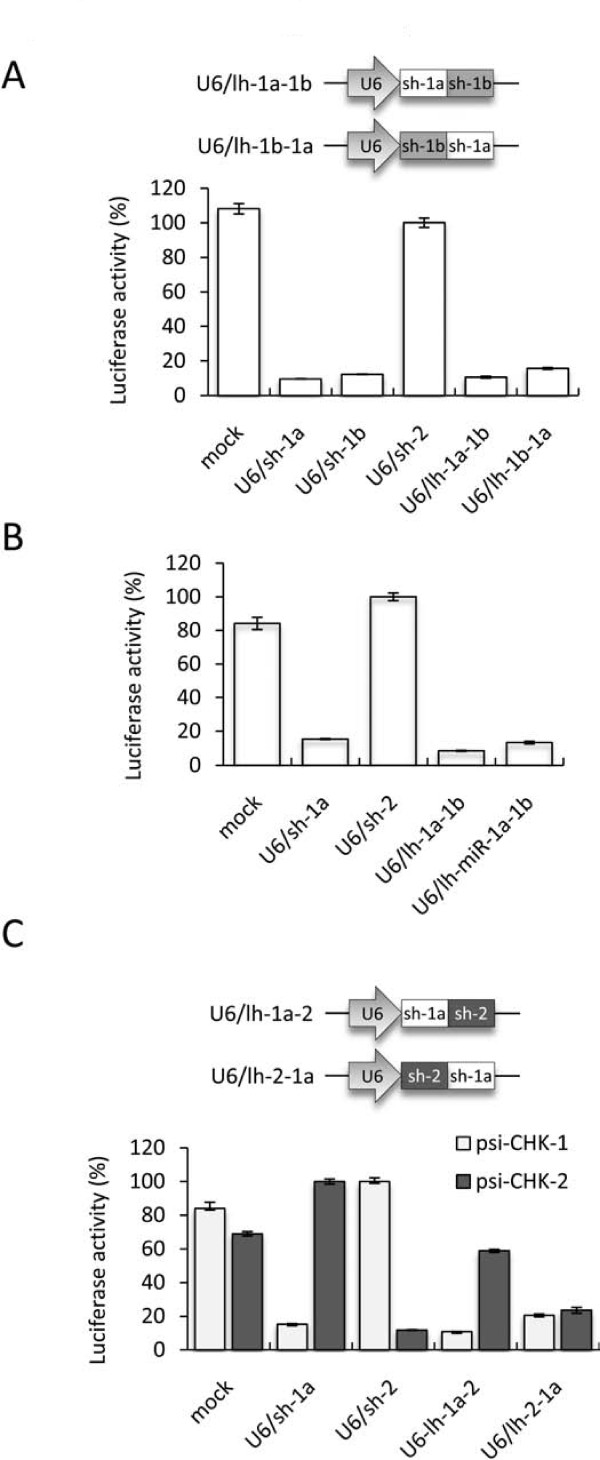
**Inhibition of Luciferase reporters by long hairpin RNAs**. (*A*) Schematic representation the single-gene targeting lhRNA plasmids, and normalised ratios of the Renilla:Firefly luciferase activity when DF-1 cells were co-transfected with psi-CHK-1 and the indicated RNAi plasmids. (*B*) Normalised ratios of the Renilla:Firefly luciferase activity when DF-1 cells were co-transfected with psi-CHK-1 and an lhRNA featuring a miR-30 loop sequence (U6/lh-miR-1a-1b), or the indicated RNAi plasmids. (*C*) Schematic representation the double-gene targeting lhRNA plasmids, and normalised ratios of the Renilla:Firefly luciferase activity when DF-1 cells were co-transfected with either psi-CHK-1 or psi-CHK-2 and the indicated RNAi plasmids. The mock control refers to a reporter plasmid alone transfection. Values are shown as percentages of the non-targeting shRNA as the mean of 4 replicates ± standard error.

Several parameters that determine effective processing of lhRNAs have been tested and have resulted in effective gene silencing and inhibition of HIV-1 replication [5,19,20]. Based on the study by Poi Liu et al., (2007) we chose to use the lhRNA organisation strategy that appeared to give the most reliable gene suppression. This consisted of 19-nt siRNA stems separated by a 3-nt spacer region, and each strand separated by the commonly used shRNA loop sequence (5'-UUCAAGAGA-3') that was originally tested by Brummelkamp et al. (2002). To see if an lhRNA could produce enhanced single-target gene suppression, two constructs were generated that encoded sh-1a and sh-1b in either the first or second positions (Figure 4A). Co-transfection of these constructs along with psi-CHK-1 showed that both lhRNA plasmids were efficient at suppressing *Renilla *expression, although this was only equal to the single U6/shRNA constructs. Despite these lhRNAs not providing an additive affect, these data further confirm that this lhRNA configuration is effective and robust, as no optimisation of shRNA or spacer sequences was required. It has been shown that shRNA efficacy can be considerably improved by the use of naturally occurring loop sequences, such as the miR-30 loop, instead of arbitrary sequences [23,31]. To test this, we inserted the miR-30 loop sequence (5'-CTGTGAAGCCACAGATGGG-3') into the most effective lhRNA construct, U6/lh-1a-1b. It was found that this did not increase the activity of the lhRNA as the level of reporter suppression was similar to the original construct (Figure 4B). To test if two separate genes could be targeted using lhRNAs and to further analyse the effect of siRNA location, we constructed vectors that encoded the sh-1a and sh-2 sequences in either of the two positions (Figure 4C). Co-transfection of these two plasmids along with either psi-CHK-1 or psi-CHK-2 resulted in effective knockdown of *Renilla *for both reporters, although the suppression by sh-2 when in the second position was considerably reduced. The most efficient lhRNA, U6/lh-2-1a, was less effective compared to the single shRNA controls, but reduced both target genes by nearly 80%. Given that sh-1a was most efficient in the first position, and sh-2 was most efficient in the second position, these data suggest that siRNA location in the lhRNA does not determine efficacy alone, but the individual properties of each siRNA is also an important determinant.

### Efficacy of single and double-gene targeting U6-expressed miRNA-embedded shRNAs

**Figure 5:**
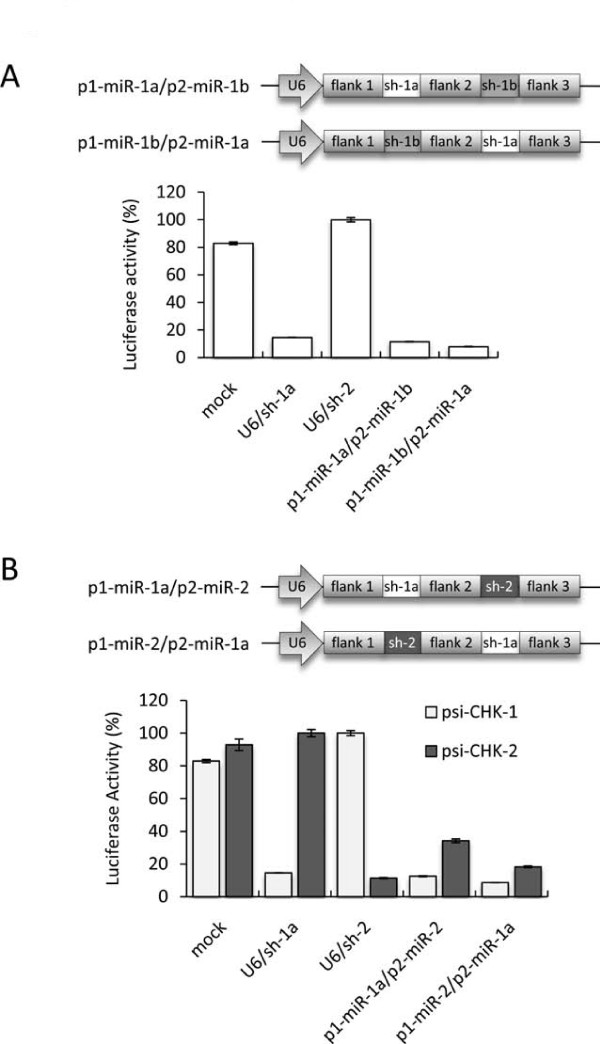
**Inhibition of Luciferase reporters by miRNA-embedded shRNAs**. (*A*) Schematic representation the single-gene targeting miRNA-embedded shRNA plasmids, and normalised ratios of the Renilla:Firefly luciferase activity when DF-1 cells were co-transfected with psi-CHK-1 and the indicated RNAi plasmids. (*B*) Schematic representation the double gene targeting miRNA-embedded shRNA plasmids, and normalised ratios of the Renilla:Firefly luciferase activity when DF-1 cells were co-transfected with either psi-CHK-1 or psi-CHK-2 and the indicated RNAi plasmids. The mock control refers to a reporter plasmid alone transfection. Values are shown as percentages of the non-targeting shRNA as the mean of 4 replicates ± standard error.

To validate the activity of shRNAs embedded into a miRNA context, dual shRNA expression constructs were generated using the vector pRFPRNAi [30]. This plasmid features sequences from the chicken miRNA operon encoding miR-106a, 18b, 20b, 19b-2, 92-2 and 363 [37] modified to contain two consecutive shRNA insertion sites. pRFPRNAi uses the chicken U6-3 promoter to transcribe shRNAs modified to mimic miR-30 and can result in effective gene suppression of both reporter and endogenous gene targets [38-40]. We first compared the activity of two pRFPRNAi vectors to target a single transcript by the expression off sh-1a and sh-1b from each of the two miRNA loci. It was found that both constructs provided knockdown to a level that was at least equal to the single shRNA vector U6/sh-1a (Figure 5A). The ability of this vector to deliver shRNAs to target two separate transcripts was then determined by inserting sh-1a and sh-2 in each of the two miRNA loci. Although both vectors effectively suppressed the two reporters simultaneously, p1-miR-2/p2-miR-1a was the most effective at reducing both targets (Figure 5B). Interestingly, these data show that the activity of sh-2 was reduced when expressed as a miRNA, a finding that was also seen when sh-2 was encoded as an lhRNA. In addition, considering that sh-1a was most efficient in position 2, and sh-2 was more effective in position 1, it appeared that shRNA location alone did not determine molecule efficacy. Overall, these data show that miRNA-embedded shRNAs can effectively suppress gene expression for both single and double gene targets, although the efficacy of sh-2 was somewhat reduced.

### Comparison of co-RNAi methods by retroviral delivery

To determine which of the three co-RNAi methods represents the most effective and robust strategy for gene suppression, we directly compared the most efficient single and double-gene knockdown vectors for their ability to suppress reporter gene activity. To avoid the inconsistencies associated with co-transfection of RNAi and reporter plasmids, and to provide a background that is more applicable to experimental gene knockdown studies, we inserted the most effective of each dual RNAi cassette into the retroviral vector RCASBP(A)-CN-EGFPm5. However, Considering that the recombination and deletion of repeated U6 sequences in viral vectors has been seen before [4,41], prior to insertion of the dual-U6 cassettes into RCAS, we swapped the second U6 promoter of these constructs with the chicken U6-4 promoter. It was found that sh-1b and sh2 expressed from this promoter achieved a similar level suppression to those transcribed by chU6-3 (data not shown), a finding that is consistent with others [33,42].

**Figure 6:**
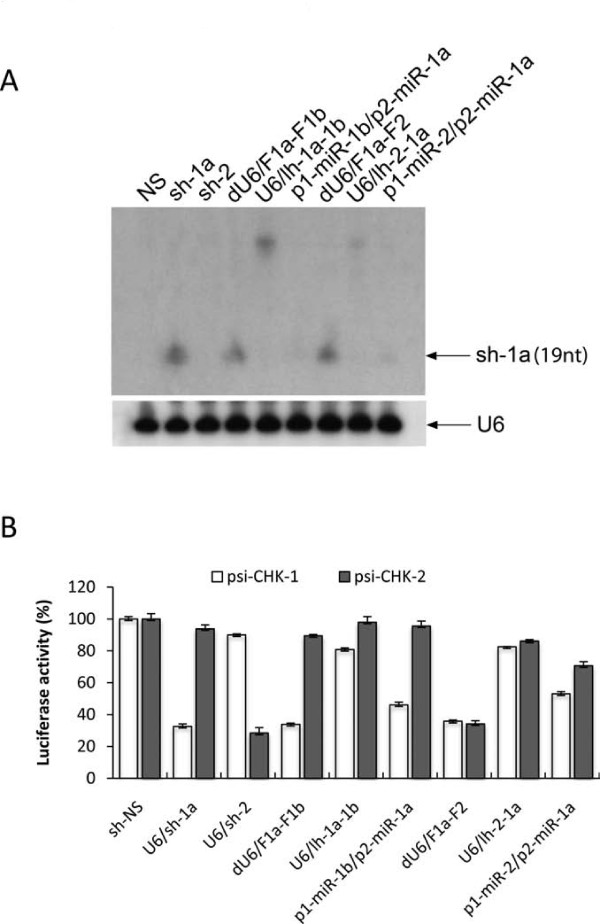
**Direct comparison of the three co-RNAi strategies by retroviral delivery**. (*A*) Northern blot analysis of total RNA extracted from DF-1 cells stably expressing single and double-gene targeting co-RNAi cassettes probed for sh-1a. (*B*) Normalised ratios of the Renilla:Firefly luciferase activity when DF-1 cells stably expressing single and double gene targeting co-RNAi cassettes were transfected with either psi-CHK-1 or psi-CHK-2. Values are shown as percentages of the negative control shRNA (sh-NS), as the mean of 4 replicates ± standard error.

Stable cell lines were produced by incubating transfected DF-1 cells for 6 days until at least 95% of were EGFP positive and were passaged into a larger vessel for RNA isolation or into 96-well plates for transfection with reporter plasmids. The expression of sh-1a was examined by Northern blot hybridization using a probe for sh-1a (Figure 6A). The positive control shRNA (sh-1a) was heavily expressed and the levels of this shRNA for the dual-U6/shRNA constructs (dU6-F1a-F1b and dU6-F1a-F2) were similar. For the lhRNA constructs, there were dense bands higher in the blot indicating high levels of unprocessed lhRNA precursors. The relative levels of the shRNA produced as miRNA-embedded transcripts were also at much lower levels compared to those expressed as regular shRNAs. As these bands were of the same size as the positive control shRNAs, this suggested that although they were effectively processed to shRNAs, they were less abundant. To directly compare the efficacy of each method of co-RNAi, each stable cell line was transfected with either psi-CHK-1 or psi-CHK-2 and relative levels of knockdown were determined by luciferase assay (Figure 6B). It was found that suppression was generally at much lower levels than seen by co-transfection, even for the single shRNA positive controls. This may be explained by the presence of more copies of the expression constructs in the plasmid transfected cells compared to the viral transduced cells. Cells transduced with the dual U6/shRNA constructs were effective to a level similar to the single shRNA controls, the lhRNAs provided only very modest knockdown, and the miRNA-embedded shRNAs were somewhat varied. Although these data were consistent with the bands visualised by Northern blotting, it was surprising to see such a decrease in knockdown efficiency compared to that achieved by the co-transfection of plasmid vectors. Overall, the dual U6-expressed shRNAs targeting both single and double gene combinations provided the most consistent knockdown, although they were unable to improve upon the single shRNA constructs alone.

### Analysis of the limitations of co-RNAi

The limitations of lhRNA-mediated RNAi are evident both in previous studies and by the data presented in the current study. It has been shown that the siRNA activity diminishes with distance from the lhRNA base [19-21], and we showed that the efficacy of the three siRNA tested varied depending on the sequence and location within the lhRNA (Figure 4A and 4C). Taken together, it appears that although the use of lhRNA can result in highly effective gene knockdown, this might require detailed optimisation for a given set of sequences, rather than simply linking effective siRNAs sequences together.

**Figure 7:**
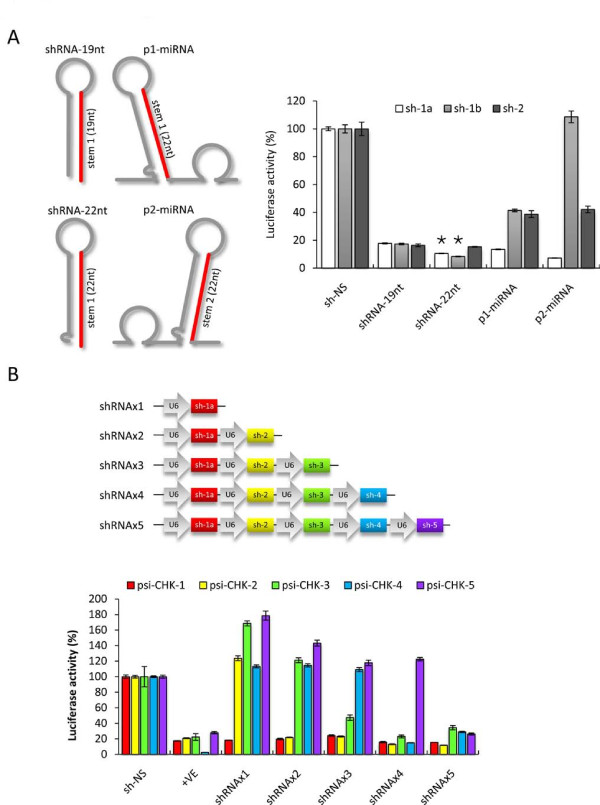
**Testing the limitations of miRNA-embedded shRNAs and multiple U6/shRNA cassettes by inhibition of Luciferase reporters**. (*A*) The *left *panel shows a schematic representation of the predicted transcription products of a standard 19-nt shRNA (shRNA-19nt), a miR-30 mimic shRNA with a 3-nt extended stem and mismatched bp in the passenger strand (shRNA-22nt), a miRNA mimic shRNA from the first locus in pRFPRNAi (p1-miRNA), and a miRNA mimic shRNA from the second locus in pRFPRNAi (p2-miRNA). For each the siRNA stem is shown in red. The *right *panel shows normalised ratios of the Renilla:Firefly luciferase activity when DF-1 cells were co-transfected with the indicated RNAi plasmids and their target Luciferase reporter plasmids (asterisks indicate P < 0.05 compared to equivalent 19-nt regular shRNAs, n = 4, two-tailed unpaired T-tests). (*B*) The *top *panel shows a schematic representation of the multiple U6/shRNA constructs featuring 1, 2, 3, 4 or 5 individual U6/shRNA cassettes. The *bottom *panel shows normalised ratios of the Renilla:Firefly luciferase activity when DF-1 cells were co-transfected with the indicated RNAi plasmids and each of the luciferase reporter plasmids. The +VE control shRNAs used for each reporter were: sh-1a for psi-CHK-1, sh-2 for psi-CHK-2, sh-3 for psi-CHK-3, sh-4 for psi-CHK-4 and sh-5 for psi-CHK-5. Values are shown as percentages of the negative control shRNA (sh-NS), as the mean of 4 replicates ± standard error.

To further investigate the versatility of the miRNA-embedded co-RNAi approach, we tested the effect of having various siRNA sequences in either of the 2 miRNA loci of pRFPRNAi. To test if the shRNAs expressed from pRFPRNAi were effective in the absence of the surrounding miRNA sequences, we constructed "miRNA mimic" shRNAs based on the miR-30 structure that encoded siRNA stems extended by 3-nt and a single mismatched nucleotide at the 5' end (Figure 7A). Co-transfection of RNAi plasmids along with appropriate reporter plasmids showed that the sh-1a and sh-1b 22-nt miRNA mimic shRNAs were significantly more effective than their equivalent 19-nt regular shRNAs (P < 0.05), whereas there appeared to be no change in the activity of the sh-2 molecule (Figure 7A). In contrast, individual shRNA efficacy varied greatly when expressed from each of the two miRNA loci. In particular, sh-1a showed increased activity in position 2, sh-1b showed reduced activity in position 1 and was completely inert in position 2, and sh-2 showed a decrease in activity for both loci. Overall, it appeared that individual shRNA efficacy could be increased by mimicking the miR-30 structure, but activity of the shRNAs expressed from pRFPRNAi varied greatly depending both on shRNA sequence and location.

In the current study, it was shown that cassette location and orientation in the dual-U6/shRNA vectors did not markedly affect shRNA activity. However, a previous report found that the abundance of individual shRNAs expressed from similar constructs decreased with when four shRNA cassettes were present [4], although the effect of this on gene suppression was not tested. To further explore these observations, we generated a series of vectors featuring increasing numbers of U6/shRNA cassettes and tested their ability to induce reporter gene knockdown (Figure 7B). A total of five U6/shRNA cassettes targeting five different reporter sequences were developed, allowing for the analysis of individual shRNA efficacy from vectors carrying 1, 2, 3, 4 or 5 separate U6/shRNA cassettes. Co-transfection of the multiple U6/shRNA plasmids along with appropriate reporter vectors showed that all of these were able to suppress gene expression at a rate similar to that of the positive controls (single U6/shRNA) regardless of how many cassettes were present. In particular, the shRNAx5 vector induced comparable levels of gene suppression to each of the single U6/shRNA positive controls, except for sh-4 however, which showed a 20% reduction in activity. A reduction in activity was also evident for the shRNAx3 vector when targeting the psi-CHK-3 reporter, and the shRNAx5 when targeting the psi-CHK-4 reporter, as both were slightly less effective compared to their respective positive control shRNAs. Importantly however, reporters 1, 2 and 5 showed equally efficient knockdown regardless of the number of shRNA cassettes present. Overall, these data suggest that vectors encoding up to five U6 promoters can maintain very high levels of individual gene silencing activities.

## Discussion

Co-RNAi has been used to achieve potent gene silencing of single and multiple-gene targets by means of a number of different techniques. Although all of these methods can result in efficient gene suppression, such studies often involve the detailed optimisation of the chosen system and experimental findings are not reported in the context of the other delivery options. Since the initial use of pol III expressed shRNAs in mammalian cells [43,44] this strategy has become a standard technique for single-target vector delivered RNAi and has been the subject of extensive optimisation. More recently, the analysis of key factors that determine lhRNA and miRNA-embedded shRNA efficacy, has seen both of these strategies achieve enhanced gene knockdown. For lhRNAs this has included siRNA length and spacing between siRNAs stems [19,20], and for miRNA-embedded shRNAs the use various flanking sequences and lengths [26,28], and the placement [29] of siRNAs within miRNA sequences contexts has been tested.

Following the identification of effective siRNA molecules for target genes of interest, the selection of a delivery strategy for co-RNAi presents several options, each of which appear to offer reliable gene knockdown. To determine which of these methods is the most effective, predictable and robust, we took a set of active siRNAs and directly compared their ability to suppress gene expression when delivered by each co-RNAi platform. Since the goal of this study was also to determine the ease at which each technique could be applied to any given set of siRNAs, we used standard 19-nt siRNAs rather than molecules that have been specifically optimised and selected for each individual expression system. This study therefore analyses the adaptability of standard siRNA sequences that would be generated by publicly available algorithms or as custom designed siRNAs to be used in each of the delivery platforms described. Prior to testing the siRNAs in the co-RNAi vectors, the expression of these sequences as regular 19-nt shRNAs resulted in equivalent gene suppression (Figure 2B), illustrating that these particular siRNAs can be effectively expressed as shRNAs without optimisation. Initially, we analysed dual shRNA expression approaches as previous experiments suggest that although using three and four shRNA expression constructs can be highly effective, these only slightly improve on the levels of gene silencing seen by the dual systems [5,29]. In addition, the levels of shRNA generated from multiple promoter constructs diminished when numerous promoters were used [4], and the second, third and fourth siRNA in lhRNA constructs become increasingly less effective the further they are away from the stem [5,19,20]. Therefore, to keep the number of variables at a minimum, this study initially involved the comparison of multiple promoter/shRNA, lhRNAs and miRNA-embedded shRNA vectors expressing only two siRNAs targeting either single or double gene combinations.

Our data showed that both dual-U6/shRNA and miRNA-embedded expression plasmids increased gene suppression of single-gene targets compared to equivalent to single shRNAs. In contrast, the single-gene targeting lhRNAs only suppressed gene expression to levels equivalent to the first siRNA, which is not surprising considering the reported gradient-effect in which they are produced. For the double-gene targeting plasmids, both lhRNA and miRNA-embedded shRNAs were more variable and showed slightly decreased gene knockdown compared to the dual-U6/shRNA plasmids, which performed equally well as single shRNAs. Using retroviral-delivered co-RNAi, each expression platform was directly compared showing that although the single-gene targeting miRNA-embedded vector was reasonably effective, it was only the dual-U6/shRNA vectors that achieved a similar level of both single and double reporter gene knockdown to the control shRNAs. However, considering that these vectors were not able to provide enhanced gene knockdown (as seen in the co-transfections in Figure 3A), overall the dual-U6/shRNA constructs did not provide a substantial increase in activity compared to single U6/shRNAs. The lack of activity of the lhRNA retroviruses was explained by the appearance of unprocessed RNA precursors by Northern blotting, and the presence of faint bands for the miRNA-embedded shRNAs was consistent with their low activity in the reporter assays. These data showed that for this set of siRNAs, despite using slightly optimised configurations for both lhRNA and miRNA-embedded shRNA, that the dual-U6/shRNAs provided the most effective and robust gene silencing.

By comparing the efficacy of different siRNA molecules expressed as lhRNAs or miRNAs, it appeared that both the location and individual properties of each siRNA could affect silencing activity. Perhaps qualities such as sequence and local structure of different molecules can directly impact upon efficient processing and production of siRNAs, possibly through changes in their thermodynamic properties. For example, when expressed as an lhRNA, sh-1a was most effective in the first position, whereas sh-2 was more effectual in the second position. Furthermore, when expressed as miRNA-embedded shRNAs, sh-1a was most efficient in position 2, whereas sh-2 which was most effective in position 1. To further explore this, we examined the individual efficacy of three different shRNAs expressed from each of the locations as embedded-miRNAs and found enormous variation ranging from increased to completely abolished activity. This suggests that the individual sequence of each shRNA can have a marked impact on the processing and subsequent activity of shRNAs expresses from each loci of this vector. Unlike lhRNAs and miRNA-embedded shRNAs, U6-transcribed regular 19-nt shRNAs do not appear to be affected by their surrounding sequences. However, since reduced levels of individual shRNAs is caused by increasing numbers of RNAi cassettes [4], we tested if this reduction translated to a functional effect on levels of gene knockdown. Overall, the expression of up to 5 separate shRNAs from a single construct resulted in a small decrease in activity for only one of the five targets, suggesting that although each shRNA might be less abundant, there was still a sufficient amount to reduce gene expression to similar levels. One concern over the use of multiple promoters for co-RNAi, and indeed strong promoters such as U6 in general, is the saturation of the RNAi machinery resulting in impaired processing of endogenous miRNAs [22,45,46]. These data would suggest however, that since pol III transcribes individual shRNAs from multiple promoter constructs at lower levels than single promoter cassettes, the overall levels of shRNAs in these cells may be similar to those containing single cassettes and may therefore not further add to this concern.

## Conclusions

The parameters that determine shRNA efficacy are now very well defined; the same cannot be said however, for constructs that express multiple RNAi effectors. By directly comparing the same set of siRNAs expressed using three separate methods of co-RNAi we have identified some of the strengths and pitfalls associated with each technique. Overall, the use of multiple U6/shRNA cassettes offered the most reliable and predictable gene knockdown of both single and multiple-gene targets, an effect that was not inhibited by having up to five separate shRNA cassettes. It remains to be seen however, the impact of having multiple pol III promoters on cellular RNAi machinery, endogenous miRNA processing and the expression of native pol III transcripts. In such a case, the use of RNAi effectors that resemble naturally occurring molecules such as miRNAs and long dsRNAs, may best avoid unwanted cellular effects, especially considering that miRNA mimic shRNAs can abolish competition of siRNAs and shRNAs for transport and incorporation into RISC [22]. In any case, this study for the first time directly compares three methods for the delivery of multiple shRNAs and provides valuable insights for the design and application of reliable combinatorial RNAi.

## Authors' contributions

LSL constructed the U6/shRNA, lhRNA, reporter vectors, and retroviral vectors, carried out all RNAi experiments, and drafted the manuscript. NJVH constructed the miRNA-embedded constructs and helped draft the manuscript. SAW developed the pRFPRNAi vectors and helped with the manuscript. VN helped conceive the study, participated in its design and coordination, and assisted with writing the manuscript. All authors read and approved the final manuscript

## Supplementary Material

Additional file 1 **List of oligonucleotides used to construct co-RNAi vectors used in this study**. Additional Table S1 - DNA oligonucleotides used to construct shRNA vectors. Additional Table S2 - DNA oligonucleotides used to construct lhRNA vectors. Additional Table S3 - DNA oligonucleotides used to construct miRNA-embedded shRNA vectors. Click here for file

Additional file 2 **Schematic depiction of the construction of multiple U6/shRNA vectors encoding up to five shRNA cassettes**. Schematic depiction of the construction of multiple U6/shRNA vectors encoding up to five shRNA cassettes. Using a series of PCR and cloning steps, consecutive U6/shRNA cassettes were inserted in a step-wise method. For each of the three steps, one additional U6/shRNA was added to the previous vector, starting with the dual-U6/shRNA construct dU6-F1a-F2. PCR fragments encoded the U6 promoter, an shRNA sequence and added unique enzyme sites. The term sh3 refers to the third U6/shRNA cassette, sh4 refers to the fourth U6/shRNA cassette and sh5 refers to the fifth U6/shRNA cassette. The name of each resultant vector is shown to left of each construct. Click here for file
