# Supplementary material for: A direct comparison of strategies for combinatorial RNA interference
Source: BMC Mol Biol. 2010 Oct 11;11:77. doi: 10.1186/1471-2199-11-77 (PMC2958852; doi:10.1186/1471-2199-11-77)
Supplement: Additional file 1 — List of oligonucleotides used to construct co-RNAi vectors used in this study. Additional Table S1 - DNA oligonucleotides used to construct shRNA vectors. Additional Table S2 - DNA oligonucleotides used to construct lhRNA vectors. Additional Table S3 - DNA oligonucleotides used to construct miRNA-embedded shRNA vectors. [file 1471-2199-11-77-S1.PDF]

Additional File 1 Lambeth et al

Table S1. DNA oligonucleotides used to construct shRNA vectors

shRNA	strand	Sequence (5'-3')
sh-1a	top	CGGCACATTTGTCGAGCTTAACTGTGAAGCCACAGATGGGTTAAGCTCGACAAATGTGCTTTTTGGAA
sh-1a	bottom	CGCGTTCCAAAAAGCACATTTGTCGAGCTTAACCCATCTGTGGCTTCACAGTTAAGCTCGACAAATGTGC
sh-1b	top	CGGGTTGGACATGTACAATATCTGTGAAGCCACAGATGGGATATTGTACATGTCCAACCTTTTTGGAA
sh-1b	bottom	CGCGTTCCAAAAAGGTTGGACATGTACAATATCCCATCTGTGGCTTCACAGATATTGTACATGTCCAACC
sh-2	top	CGGAGTTATGCTGATATGAATCTGTGAAGCCACAGATGGGATTCATATCAGCATAACTCTTTTTGGAA
sh-2	bottom	CGCGTTCCAAAAAGAGTTATGCTGATATGAATCCCATCTGTGGCTTCACAGATTCATATCAGCATAACTC
sh-3	top	CGGGAGTTCACTGTATCGTACCTGTGAAGCCACAGATGGGGTACGATACAGTGAACTCCTTTTTT
sh-3	bottom	CGCGAAAAAAGGAGTTCACTGTATCGTACCCCATCTGTGGCTTCACAGGTACGATACAGTGAACTCC
sh-4	top	CGGCTGGACTCCTTCATCAACCTGTGAAGCCACAGATGGGGTTGATGAAGGAGTCCAGCTTTTTT
sh-4	bottom	CGCGAAAAAAGCTGGACTCCTTCATCAACCCCATCTGTGGCTTCACAGGTTGATGAAGGAGTCCAGC
sh-5	top	CGCAGCCAATCACATCCATCAAACTGTGAAGCCACAGATGGGTTTGATGGATGTGATTGGCTGTTTTTT
sh-5	bottom	CGCGAAAAAACAGCCAATCACATCCATCAAACCCATCTGTGGCTTCACAGTTTGATGGATGTGATTGGCTG
sh-1a-22nt	top	CGTCACATTTGTCGAGCTTAATCTTAGTGAAGCCACAGATGTAAGATTAAGCTCGACAAATGTGCTTTTTT
sh-1a-22nt	bottom	CGCGAAAAAAGCACATTTGTCGAGCTTAATCTTACATCTGTGGCTTCACTAAGATTAAGCTCGACAAATGTGA
sh-1b-22nt	top	TGTTGGACATGTACAATATTTCTAGTGAAGCCACAGATGTAGAAATATTGTACATGTCCAACCTTTTTT
sh-1b-22nt	bottom	CGCGAAAAAAGGTTGGACATGTACAATATTTCTACATCTGTGGCTTCACTAGAAATATTGTACATGTCCAACA
sh-2-22nt	top	CGTAGTTATGCTGATATGAATTACTAGTGAAGCCACAGATGTAGTAATTCATATCAGCATAACTCTTTTTT
sh-2-22nt	bottom	CGCGAAAAAAGAGTTATGCTGATATGAATTACTACATCTGTGGCTTCACTAGTAATTCATATCAGCATAACTA
sh-NS	top	CGTTCTCCGAACGTGTCACGTCTGTGAAGCCACAGATGGGACGTGACACGTTCGGAGAATTTTTGGAA
sh-NS	bottom	CGCGTTCCAAAAATTCTCCGAACGTGTCACGTCCCATCTGTGGCTTCACAGACGTGACACGTTCGGAGAA

Table S2. DNA oligonucleotides used to construct lhRNA vectors

**lhRNA**	**Strand**	**Sequence (5'-3')**
lh-1a-1b	Top	CGGCACATTTGTCGAGCTTAATCTGGTTGGACATGTACAATATTTCAAGAGAATATTGTACATGTCCAACCAGATTAAGCTCGACAAATGTGCTTTTTT
lh-1a-1b	Bottom	CGCGAAAAAAGCACATTTGTCGAGCTTAATCTGGTTGGACATGTACAATATTCTCTTGAAATATTGTACATGTCCAACCAGATTAAGCTCGACAAATGTGC
lh-1b-1a	Top	CGGGTTGGACATGTACAATATTTCGCACATTTGTCGAGCTTAATTCAAGAGATTAAGCTCGACAAATGTGCGAAATATTGTACATGTCCAACCTTTTTT
lh-1b-1a	Bottom	CGCGAAAAAAGGTTGGACATGTACAATATTTCGCACATTTGTCGAGCTTAATCTCTTGAATTAAGCTCGACAAATGTGCGAAATATTGTACATGTCCAACC
lh-1a-2	Top	CGGCACATTTGTCGAGCTTAATCTGAGTTATGCTGATATGAATTTCAAGAGAATTCATATCAGCATAACTCAGATTAAGCTCGACAAATGTGCTTTTTT
lh-1a-2	Bottom	CGCGAAAAAAGCACATTTGTCGAGCTTAATCTGAGTTATGCTGATATGAATTCTCTTGAAATTCATATCAGCATAACTCAGATTAAGCTCGACAAATGTGC
lh-2-1a	Top	CGGAGTTATGCTGATATGAATTACGCACATTTGTCGAGCTTAATTCAAGAGATTAAGCTCGACAAATGTGCGTAATTCATATCAGCATAACTCTTTTTT
lh-2-1a	Bottom	CGCGAAAAAAGAGTTATGCTGATATGAATTACGCACATTTGTCGAGCTTAATCTCTTGAATTAAGCTCGACAAATGTGCGTAATTCATATCAGCATAACTC
lh-miR-1a-1b	Top	CGGCACATTTGTCGAGCTTAATC**A**GGTTGGACATGTACAATATCTGTGAAGCCACAGATGGGATATTGTACATGTCCAACCAGATTAAGCTCGACAAATGTGCTTTTT
lh-miR-1a-1b	Bottom	CGCGAAAAAGCACATTTGTCGAGCTTAATCTGGTTGGACATGTACAATATCCCATCTGTGGCTTCACAGATATTGTACATGTCCAACCTGATTAAGCTCGACAAATGTGC

Table S3. DNA oligonucleotides used to construct miRNA-embedded shRNA vectors

construct	Sequence (5’-3’)
UHP1F	GGCGGGGCTAGCTGGAGAAGATGCCTTCCGGAGAGGTGCTGCCAGTGAGCG
UHP1R	GGGTGGACGCGTAAGAGGGGAAGAAAGCTTCTAACCCCGCTATTCACCACCACCAGTAGGCA
UHP2F	GGCGGGACGCGTGCTGTGAAGATCCGAAGATGCCTTGCGCTGGTTCCTCCGCAGTGAGCG
UHP2R	CGCCGCGCATGCACCAAGCAGAGCAGCCTGAAGACCAGCAGTAGGCA
p1-miR-1aF	AGGTGCTGCCAGTGAGCGTCACATTTGTCGAGCTTAATCTTAGTGAAGCCACAGATGTA
p1-miR-1aR	CACCACCACCAGTAGGCAGCACATTTGTCGAGCTTAATCTTACATCTGTGGCTTCACT
p2-miR-1aF	GTTCCTCCGCAGTGAGCGTCACATTTGTCGAGCTTAATCTTAGTGAAGCCACAGATGTA
p2-miR-1aR	GAAGACCAGCAGTAGGCAGCACATTTGTCGAGCTTAATCTTACATCTGTGGCTTCACT
p1-miR-1bF	AGGTGCTGCCAGTGAGCGTGTTGGACATGTACAATATTTCTAGTGAAGCCACAGATGTA
p1-miR-1bR	CACCACCACCAGTAGGCAGGTTGGACATGTACAATATTTCTACATCTGTGGCTTCACT
p2-miR-1bF	GTTCCTCCGCAGTGAGCGTGTTGGACATGTACAATATTTCTAGTGAAGCCACAGATGTA
p2-miR-1bR	GAAGACCAGCAGTAGGCAGGTTGGACATGTACAATATTTCTACATCTGTGGCTTCACT
p1-miR-2F	AGGTGCTGCCAGTGAGCGTAGTTATGCTGATATGAATTACTAGTGAAGCCACAGATGTA
p1-miR-2R	CACCACCACCAGTAGGCAGAGTTATGCTGATATGAATTACTACATCTGTGGCTTCACT
p2-miR-2F	GTTCCTCCGCAGTGAGCGTAGTTATGCTGATATGAATTACTAGTGAAGCCACAGATGTA
p2-miR-2R	GAAGACCAGCAGTAGGCAGAGTTATGCTGATATGAATTACTACATCTGTGGCTTCACT
