# Supplementary material for: A direct comparison of strategies for combinatorial RNA interference
Source: BMC Mol Biol. 2010 Oct 11;11:77. doi: 10.1186/1471-2199-11-77 (PMC2958852; doi:10.1186/1471-2199-11-77)
Supplement: Additional file 2 — Schematic depiction of the construction of multiple U6/shRNA vectors encoding up to five shRNA cassettes. Schematic depiction of the construction of multiple U6/shRNA vectors encoding up to five shRNA cassettes. Using a series of PCR and cloning steps, consecutive U6/shRNA cassettes were inserted in a step-wise method. For each of the three steps, one additional U6/shRNA was added to the previous vector, starting with the dual-U6/shRNA construct dU6-F1a-F2. PCR fragments encoded the U6 promoter, an shRNA sequence and added unique enzyme sites. The term sh3 refers to the third U6/shRNA cassette, sh4 refers to the fourth U6/shRNA cassette and sh5 refers to the fifth U6/shRNA cassette. The name of each resultant vector is shown to left of each construct. [file 1471-2199-11-77-S2.PDF]

Additional File 2.
Lambeth et al. Strategy for the construction of shRNA vectors

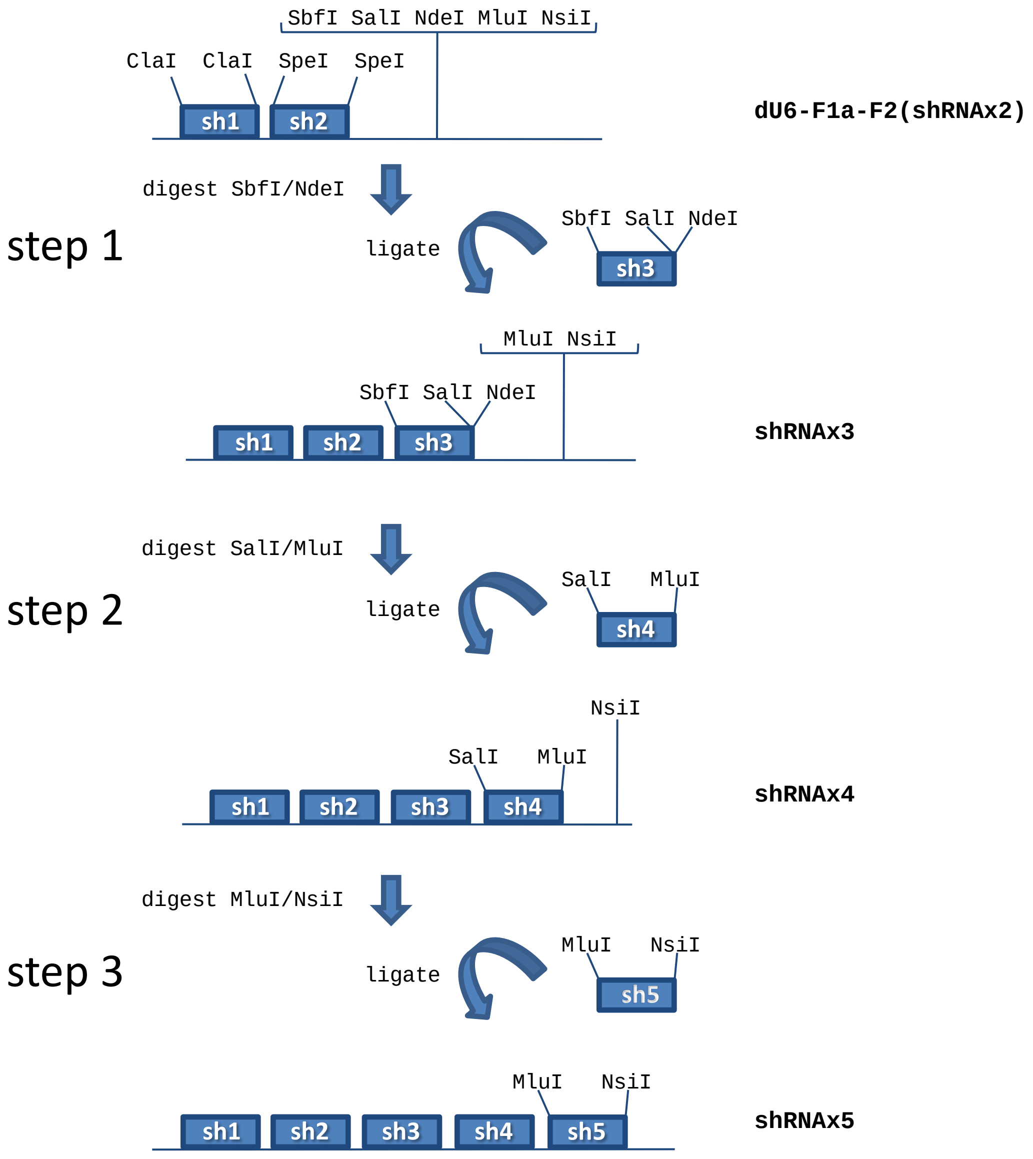

Schematic depiction of the construction of multiple U6/shRNA vectors encoding up to five shRNA cassettes. Using a series of PCR and cloning steps, consecutive U6/shRNA cassettes were inserted in a step-wise method. For each of the three steps, one additional U6/shRNA was added to the previous vector, starting with the dual-U6/shRNA construct dU6-F1a-F2. PCR fragments encoded the U6 promoter, an shRNA sequence and added unique enzyme sites. The term sh3 refers to the third U6/shRNA cassette, sh4 refers to the fourth U6/shRNA cassette and sh5 refers to the fifth U6/shRNA cassette. The name of each resultant vector is shown to left of each construct.
